# Repeated tender point injections of granisetron alleviate chronic myofascial pain - a randomized, controlled, double-blinded trial

**DOI:** 10.1186/s10194-015-0588-3

**Published:** 2015-12-03

**Authors:** Nikolaos Christidis, Shahin Omrani, Lars Fredriksson, Mattias Gjelset, Sofia Louca, Britt Hedenberg-Magnusson, Malin Ernberg

**Affiliations:** Department of Dental Medicine, Section for Orofacial Pain and Jaw Function, Karolinska Institutet, and the Scandinavian Center for Orofacial Neurosciences (SCON), Box 4064, Huddinge, SE-141 04 Sweden; Department of Clinical Oral Physiology at the Eastman Institute, Stockholm Public Dental Health, Dalagatan 11, Stockholm, SE-113 24 Sweden

**Keywords:** Granisetron, 5-HT_3_-receptor, Myofascial pain, Temporomandibular disorders, Human

## Abstract

**Background:**

Serotonin (5-HT) mediates pain by peripheral 5-HT_3_-receptors. Results from a few studies indicate that intramuscular injections of 5-HT_3_-antagonists may reduce musculoskeletal pain. The aim of this study was to investigate if repeated intramuscular tender-point injections of the 5-HT_3_-antagonist granisetron alleviate pain in patients with myofascial temporomandibular disorders (M-TMD).

**Methods:**

This prospective, randomized, controlled, double blind, parallel-arm trial (RCT) was carried out during at two centers in Stockholm, Sweden. The randomization was performed by a researcher who did not participate in data collection with an internet-based application (www.randomization.com). 40 patients with a diagnose of M-TMD according to the Research Diagnostic Criteria for Temporomandibular Disorders (RDC/TMD) were randomized to receive repeated injections, one week apart, with either granisetron (GRA; 3 mg) or isotonic saline as control (CTR).

**Results:**

The median weekly pain intensities decreased significantly at all follow-ups (1-, 2-, 6-months) in the GRA-group (Friedman test; *P* < 0.05), but not in the CTR-group (Friedman-test; *P* > 0.075). The numbers needed to treat (NNT) were 4 at the 1- and 6-month follow-ups, and 3.3 at the 2-month follow-up in favor of granisetron.

**Conclusions:**

Repeated intramuscular tender-point injections with granisetron provide a new pharmacological treatment possibility for myofascial pain patients with repeated intramuscular tender-point injections with the serotonin type 3 antagonist granisetron. It showed a clinically relevant pain reducing effect in the temporomandibular region, both in a short- and long-term aspect.

**Trial registration:**

European Clinical Trials Database 2005-006042-41 as well as at Clinical Trials NCT02230371.

## Background

Musculoskeletal pain is the most common cause of reduced work capacity and sick leave, and as result a significant health problem. Already in 2003, the annual yearly costs in Europe were estimated to be 34 billion Euro [1]. The impact of musculoskeletal pain is not only the unpleasant sensory experience but also an emotional experience with feelings of failure, misery, guilt, alienation, and co-morbid depression [2, 3]. Temporomandibular disorders (TMD) is a collective term embracing chronic pain conditions affecting the temporomandibular joint (TMJ) or the masticatory muscles (myofascial TMD; M-TMD) and their associated structures [4]. TMD has a prevalence of approximately 10–20 % and is 1.5 to 2 times more prevalent in women [5–7]. It is often associated with restricted mouth opening capacity, pain upon chewing, muscle soreness and headache, thus affecting quality of life considerably although it is not life threatening.

The neurotransmitter serotonin (5-hydroxytryptamine; 5-HT) is an important component of the chemical milieu during inflammation [8]. It is found in high concentrations in platelets, enterochromaffin cells, and in certain regions of the brain [9]. Peripherally, it is released from platelets and mast cells due to tissue damage or ischemia. 5-HT concentrations have been found to be significantly elevated in painful muscles of patients with chronic myalgia [10–12]. Evidence suggests that 5-HT activates the 5-HT_3_-receptor to mediate muscle pain and mechanical sensitivity [13–15]. In a recent study it was shown that the 5-HT_3A_-receptor is highly expressed in human masseter muscles and that more nerve fibers express 5-HT_3A_-receptors in women with M-TMD compared to healthy women [16]. Therefore, targeting peripheral 5-HT_3_-receptors with drugs that block these receptors could be an interesting therapeutic approach for chronic muscle pain.

Indeed, several specific 5-HT_3_-antagonists have been tested experimentally and clinically for treatment of muscle pain. Experimentally, systemic administration of granisetron increased the pressure pain threshold (PPT) over healthy muscles [17], while local administration reduced experimental pain and allodynia [14, 18]. Systemic administration of tropisetron had a positive analgetic effect in fibromyalgia [19, 20], whereas ondansetron reduced postoperative pain and enhanced the postoperative analgesic effect of paracetamol [21]. However, systemic administration of 5-HT_3_-antagonists is often associated with bothersome constipation which may affect treatment compliance. For localized pain conditions local administration has therefore been used with positive results [22, 23]. Even if these studies were not placebo-controlled, the combined results indicate that 5-HT_3_-antagonists may be effective to use as a new, additional therapeutic approach for both acute and chronic muscle pain. In our knowledge, no randomized controlled study (RCT) has yet investigated the long-term effect of local administration of 5-HT_3_-antagonists in chronic pain-conditions.

Consequently, this study was designed to investigate if local treatment with repeated intramuscular tender-point injections of granisetron could be effective in alleviating pain in patients with chronic M-TMD. The hypothesis was that local treatment with granisetron would be superior to isotonic saline (control-substance) regarding the outcome domains recommended for pain trials by the Initiative on Methods, Measurement, and Pain Assessment in Clinical Trials (IMMPACT) [24, 25].

## Methods

### Patients

**Fig. 1:**
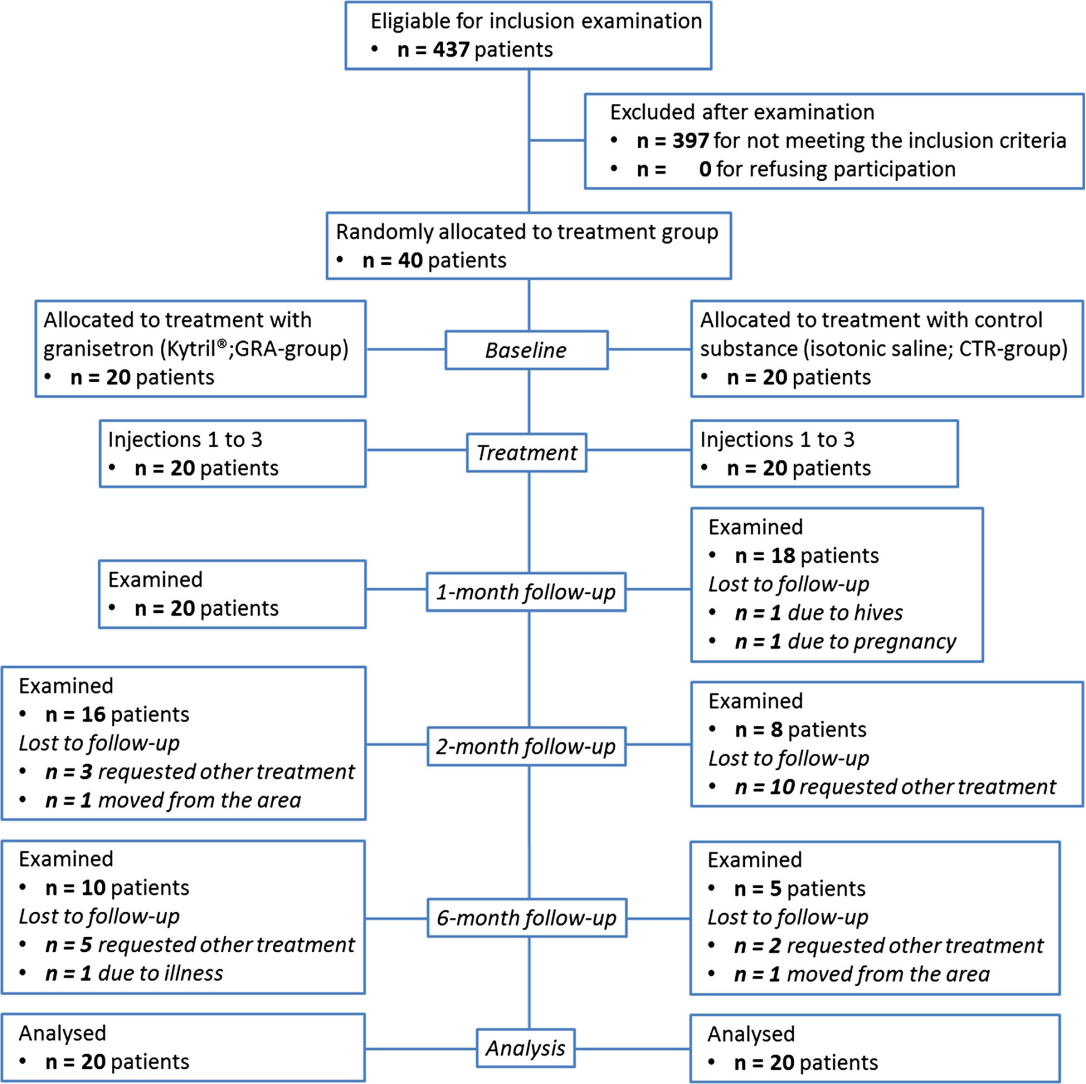
CONSORT flowchart. Flowchart of the 40 participating patients throughout the study. The patients were referred for treatment of myofascial temporomandibular disorders

Based on the information given on 1753 referrals for TMD, 437 patients with a main complaint M-TMD pain were subjected to a screening by the principal investigator (N.C.) who enrolled the patients. Forty patients (37 women and 3 men) were found eligible and included in this study; none declined participation (Fig. 1). According to the power calculation based on a previous study [26], inclusion of 17 patients in each group would be sufficient to detect a statistically significant difference of 30 % (SD 30 %), between interventions at a significance level of 5 % with a power of 80 %. In order to compensate for dropouts three additional patients were included in each group.

The inclusion criteria for the patients were; 1) age ≥ 18 years, 2) a diagnosis of myofascial pain according to the Research Diagnostic Criteria for TMD Axis I (RDC/TMD) [27], 3) duration of TMD-pain ≥3 months, 4) self-assessed average M-TMD pain intensity of ≥ 30 mm on a 100-mm visual analogue scale (VAS) during one week prior to examination, and 5) pain upon digital palpation of the masseter and/or the temporalis muscles. The patients remained included with one or several co-diagnoses of; a) disc displacement with or without reduction [27], b) osteoarthrosis [27] in the any of the TMJs, and c) tension type headache [28].

The exclusion criteria for the patients were; 1) diagnosed systemic muscular or joint diseases (e.g. fibromyalgia, rheumatoid arthritis, ankylosing spondylitis, psoriatic arthritis), 2) whiplash associated disorder, 3) neuropathic pain or neurological disorders (e.g. myasthenia gravis, orofacial dystonia), 4) history of psychiatric disorders, 5) pain of dental origin, 6) use of muscle relaxants or any medication that might influence the response to pain, 7) pregnancy or lactation, and 8) known hypersensitivity to granisetron.

The study was approved by the regional ethical review board in Stockholm, Sweden (2006/192-31/4) and by the Medical Products Agency in Uppsala, Sweden (151:2006/7947), registered in the European Clinical Trials Database (2005-006042-41) as well as at ClinicalTrials.gov (NCT02230371). The study was carried out in compliance with the Declaration of Helsinki as well as the International Conference on Harmonisation Guideline for Good Clinical Practice. All participants received both written and verbal information prior to inclusion and gave their verbal and written consent.

### Study protocol

This prospective, randomized, controlled, double blind, parallel-arm trial (RCT) follows the consolidated standards of reporting trials (CONSORT) statement [29, 30] and was carried out during the period of May 2006 to December 2014 at two centers, the specialist clinics for Orofacial Pain and Jaw Function, Department of Dental Medicine, Karolinska Institutet, Huddinge, Sweden and Clinical Oral Physiology at the Eastman Institute, Stockholm Public Dental Health (Folktandvården Stockholms län AB), Stockholm, Sweden.

The patients were divided randomly into two groups that received either granisetron or control substance. The randomization was performed by a researcher (ME) who did not participate in data collection with an internet-based application (www.randomization.com). The program generated a numbered list with the substances in random order in blocks of four. The list was kept hidden to the examiners during the entire study and the block size as well as substances not revealed until the last patient had undergone the last follow-up. The study substances have identical appearance, smell and viscosity so both the patients and examiners were blinded to group assignment. A research assistant assigned the patients to treatment substance in consecutive order according to the randomization list, prepared the syringes and marked them with patient number. Three orofacial pain specialists (NC, LF, BHM) and two DDS undergoing specialist training in orofacial pain (SO, SL), who were repeatedly calibrated to a gold standard examiner (ME) in the RDC/TMD examination during one day, performed the study.

The study comprised seven visits; V1) Screening for study participation and inclusion, V2-V4), Injection of study substances, and V5) Follow-ups at 1-month. Those patients in any group that still reported more than 30 % improvement at the 1-month visit follow-up were scheduled for follow-ups also after 2- (V6) and 6-months (V7), Fig. 1.

### Treatment

#### Treatment substances

Granisetron (KYTRIL®; 1 mg/mL, Roche, Stockholm, Sweden) was used as active treatment (GRA-group) and isotonic saline (NaCl; 0.9 mg/mL, Fresenius Kabi, Uppsala, Sweden) was used as control treatment (CTR-group). Granisetron is used to treat chemotherapy-induced and postoperative nausea and vomiting and is among the drugs that exhibit the most selective binding to the 5-HT_3_-receptor, with an affinity to the 5-HT_3_-receptor that is up to 40,000-fold of any other receptor. Granisetron is not dependent on the isoenzyme cytochrome P450 2D6 (CYP2D6), possibly leading to a good efficacy also in ultrarapid metabolizers and has, unlike the other 5-HT_3_-receptor antagonists, a long half-life [31–33]. It is considered efficacious and safe with only few, passing side-effects. The chosen solution was slowly injected into a maximum of six muscle sites in each patient. The most painful tender-points to palpation of the masticatory muscles were chosen, maximum 3 per muscle. The injected volume into each tender-point was 0.5 mL. Thus the maximum dose of granisetron a patient could receive was 3 mg per treatment. The dose is equivalent to the dose of the 5-HT_3_-receptor antagonist tropisetron used for trigger point injections in similar studies [23, 26, 34].

The injections were made perpendicular to the skin surface over the chosen tender-point with an angle of 90° using a 19-mm long needle (diameter 0.4 mm) from a 2-mL syringe. The solution was administered into each tender-point as a single shot during 10 s. In order to ensure intramuscular administration, during the study, the injections were guided by electromyography (Allergan AB, Stockholm, Sweden) in order to ensure intramuscular administration. The injections were repeated after 1- and 2-weeks in the most painful tender-points at that time.

#### Follow-ups

Follow-ups were performed by questionnaires, pain assessment, clinical examination and recordings of PPT. Patients who did not respond to treatment were offered additional treatment at the 1-month follow-up and were excluded from further analyses. They were thus regarded as non-responders in the intention-to-treat-analyses (ITT-analyses), Fig. 1.

### Treatment outcome measures

The IMMPACT recommends six core domains to be reported in RCTs; participant disposition, pain intensity, physical functioning, emotional functioning, participant ratings of overall improvement and adverse events [24, 25]. The change in weekly pain intensity from baseline was the primary outcome measure. The other domains recommended by IMMPACT served as secondary outcome measures.

Additional outcome measures were changes in PPT, 5-HT platelet poor plasma (P-5-HT), as well as pain distribution.

#### Pain characteristics

Pain intensity was retrieved from the 1-week pain diary. This was composed of seven 0–100 mm visual analogue scales (VAS) with the endpoints marked “no pain” (0) and “worst experienced pain” (100) for daily assessments of the average orofacial pain intensity the week preceding each visit. For each patient the average pain intensity during that week was calculated (called weekly pain intensity). Several repeated assessments are shown to be a more valid measure of pain intensity than a single assessment [35]. The present study reports both 30 % and 50 % pain reduction, since ≥30 % and ≥50 % reductions of chronic pain respectively reflect a clinically relevant pain reduction and substantial improvement [36]. The patients further marked their pain distribution on two lateral views of the head, one for each side separately.

#### Physical functioning

Physical functioning was classified using the Graded Chronic Pain Scale (GCPS) [37] and the Jaw Disability Checklist (JDC), both included in the RDC/TMD Axis II questionnaire [27]. The GCPS severity scale is divided into two parts. The first part is used to assess characteristic pain intensity and the second part limitations in physical functioning due to pain. When assessing physical functioning a disability score (DP 0–6) is combined with pain intensity (0–100) as follows: Grade 0 = no TMD-pain in the previous 6 months; Grade I = low disability (<3 DP) and low intensity pain (<50); Grade II = low disability (<3 DP) and high intensity pain (>50); Grade III = high disability, moderately limiting (3–4 DP regardless of pain intensity); Grade IV = high disability, severely limiting (5–6 DP regardless of pain intensity).

JDC was used for assessment of limitation in jaw function. The JDC consists of twelve questions assessing to which extent TMD interferes with activities specifically related to the mandibular function. It has a maximum of 12 points where 0 = no limitation in any mandibular function and 12 = limitations in all mandibular functions.

In addition, the pain-free mouth opening and the maximum voluntary mouth opening (MVO), including the vertical overbite, was assessed in millimeters with a ruler.

#### Emotional functioning

The changes in emotional functioning were assessed using the modified SCL-90-R instrument in the RDC/TMD Axis II questionnaire [27]. This includes twenty questions indicating depression and 12 questions indicating nonspecific physical symptoms (NSPhS). The total score is calculated (graded: 0–4). The degree of depression was classified as normal (<0.535), moderate (0.535-1.105) or severe (>1.105), while the classification for NSPhS is: normal (<0.5), moderate (0.5-1) and severe (>1).

#### Overall improvement

The patients overall improvement was assessed on a 6-point rating scale: 0 = symptom-free; 1 = much better; 2 = better; 3 = unchanged; 4 = worse; 5 = much worse. This scale is commonly used and validated [36, 38].

#### Adverse events

In order to estimate any adverse events the patients were asked to list any adverse event during the week following each injection. If any adverse event occurred they were asked to describe the event and to grad it as mild, moderate or severe.

#### Assessment of pressure pain threshold

To assess the PPT an electronic pressure algometer (Somedic Sales AB, Hörby, Sweden) was used. This device consists of a grip with a pressure-sensitive strain gauge at the tip and a display unit. The 1 cm^2^ probe tip was covered with a 1 mm thick rubber pad to minimize the risk of irritation of the skin. The algometer was held perpendicular to the skin surface over the muscles and the pressure was increased at a standardized rate of 50 kPa per s. The patients were instructed to press a signal button when the sensation of “pressure” changed into “pain”. This was first performed over the soft tissue close to the base of the thumb on the dorsal side of the right hand, in order to accustom the subject to the procedure. Recordings of PPT were made bilaterally over the most prominent points of the masseter muscles as well as over a reference point on the tip of the right index finger. An extra-cranial reference point was chosen to investigate any possible systemic effects by the study treatments. When assessing the PPT the mean value of 3 measurements with an interval of 2 min was used.

#### Blood sampling

A blood sample (5 mL) was collected from the decubital vein in EDTA-containing tubes for P-5-HT. The sample was immediately cooled and centrifuged (1700 G) for 30 min. Approximately 200 μL of the supernatant was then collected with a pipette into polystyrene tubes and kept frozen at −80° until the analysis. The plasma samples were analyzed in our laboratory with a commercial EIA-Serotonin kit (No IM1749, Immunotech, Beckman Coulter, Marseille, France). The kit has a sensitivity of 0.5 nM, and according to the manufacturer it has an intra-assay coefficient of variation between 8.9 % and 14.5 %, and an inter-assay coefficient variation between 9.9 % and 11.5 %.

The patients were asked to avoid tryptophan-rich food (e.g. eggs, milk, chicken, turkey, tomato, banana, pineapple, and chocolate) for 24 h before the examination to exclude any risk of interference with the analysis of 5-HT.

### Statistical analyses

The statistical analyses were performed using SigmaPlot software version 13.0 (Systat software Inc., San Jose, CA, USA). Normality of the data was tested with the Shapiro-Wilk test. Descriptive data are reported as frequencies, means and standard deviation (SD) or median and interquartile range (IQR). For analyses of group differences in frequencies *χ*^2^-test was used. For analyses of group differences of variables on a nominal scale *t*-test was used, while the Mann–Whitney *U*-test was used for variables on an ordinal scale. The Friedman’s analysis of variance (ANOVA) on ranks with Dunn’s test as post hoc test was used for analyses of changes between baseline data and follow-up measurements regarding pain variables and emotional functioning, while 2-way repeated measures ANOVA with Holm-Sidak as post hoc test was used for physical functioning and changes in PPT. The Pearson correlation-test with Bonferroni-correction for multiple comparisons was used to correlate the P-5-HT levels with baseline and outcome variables. The significance level was set at *P* < 0.05.

## Results

The results are presented for both patient centers combined (Karolinska Institutet, Huddinge, Sweden and Eastman Institute, Stockholm Public Dental Health (Folktandvården Stockholms län AB), Stockholm, Sweden) since there were no significant differences for any of the study outcomes between them.

There were no differences between the active treatment group (GRA-group) or the control group (CTR-group) regarding demographic data (Table 1).

**Table 1:** Demographic data of 40 patients with myofascial temporomandibular disorders randomized to treatment with active substance (GRA) or control substance (CTR)

	GRA (*n* = 20)	CTR (*n* = 20)
Sex		
Female	18	19
Male	2	1
Age		
Mean (SD)	38.3 (15.1)	39.1 (16.1)
<20	1	1
20-40	11	12
>40	8	7
Ethnic origin		
Scandinavia	14	15
Other European countries	2	3
Africa	1	0
Asia	2	1
South America	1	1
Marital status		
Never married	8	6
Married	9	12
Divorced	3	2
Highest level of education		
Elementary school	2	0
High school	9	10
College	9	10

*GRA* Granisetron (Kytril®; 1 mg/mL, Roche, Stockholm, Sweden), *CTR* control substance (isotonic saline (NaCl); 0.9 mg/mL, Fresenius Kabi, Uppsala, Sweden). There were no significant group differences

Further, there were no significant differences between the groups regarding baseline pain variables or self-reported clenching/grinding (Table 2). All patients reported moderate to severe tenderness to palpation of the masseter and/or temporalis muscles. The patients’ distribution of diagnoses according to RDC/TMD Axis I are presented in Table 2. All patients had a diagnosis of myofascial pain. Most patients (87 %) also had other TMD diagnoses. No patient used any kind of centrally acting medication prior to or during the study.

**Table 2:** Baseline pain variables, awareness of parafunctions and diagnoses according to RDC/TMD Axis I in 40 patients with myofascial temporomandibular disorders randomized to treatment with active substance (GRA) or control substance (CTR)

	GRA (*n* = 20)	CTR (*n* = 20)
RDC/TMD Axis II		
Duration of myofascial pain (months)		
Mean (SD)	80 (54)	82 (66)
6-12 months	1	1
≥12 months	19	19
Frequency of myofascial pain		
Recurrent	5	8
Persistent	15	12
Current myofascial pain intensity (NRS 0–10)		
Median (IQR)	6.0 (4.0)	6.0 (4.0)
Worst myofascial pain intensity last 6 months (NRS 0–10)		
Median (IQR)	8.0 (2.0)	8.0 (2.0)
Average myofascial pain intensity last 6 months (NRS 0–10)		
Median (IQR)	5.0 (3.0)	6.0 (5.0)
Pain area (AU)		
Median (IQR)	5930 (5059)	3861 (13596)
Awareness of clenching/grinding		
Daytime	1	2
Nighttime	2	1
Both day- and nighttime	15	15
RDC/TMD Axis I		
Myofascial pain (Ia)	10	13
Myofascial pain (Ib)	10	7
Disc displacement with reduction (IIa)		
One side	5	5
Both sides	7	7
Arthralgia (IIIa)		
One side	1	0
Both sides	4	3
Osteoarthrosis (IIIc)		
One side	0	1
Both sides	1	1

*GRA* Granisetron (Kytril®; 1 mg/mL, Roche, Stockholm, Sweden), *CTR* control substance (isotonic saline (NaCl); 0.9 mg/mL, Fresenius Kabi, Uppsala, Sweden), *RDC/TMD* Research Diagnostic Criteria for temporomandibular disorders, *SD* Standard deviation, *IQR* Interquartile range (the 75^th^ percentile minus the 25^th^ percentile), *NRS* Numeric Rating Scale, *AU* Arbitrary units; There were no significant differences between groups

There were no significant differences in mean (SD) P-5-HT levels between the groups at baseline (GRA: 43.5 (66.3); CTR-group: 97.4 (131.4) nmol/L).

Finally, no differences in demographic data, pain intensity, physical or emotional functioning and P-5-HT values were found between patients who completed the study and the drop-outs, and there were no differences in baseline variables between responders and non-responders to treatment.

### Primary treatment outcome

The median weekly pain intensities are presented in Table 3. The median weekly pain intensities had decreased significantly at all follow-ups in the GRA-group (Friedman test; *P* < 0.05), but not in the CTR-group (Friedman-test; *P* > 0.075).

**Table 3:** Pain and physical functioning before (baseline) and after (1-, 2- and 6-month follow-ups) repeated tender-point injections with active substance (GRA) or control substance (CTR), in 40 patients with myofascial temporomandibular disorders

	Baseline		1 month		2 months		6 months	
	GRA	CTR	GRA	CTR	GRA	CTR	GRA	CTR
Pain variables	*n* = 20	*n* = 20	*n* = 20	*n* = 18	*n* = 16	*n* = 8	*n* = 10	*n* = 5
Pain intensity	52 (29)	57 (24)	29 (41)^a^	29 (40)	32 (30)^ab^	36 (25)	24 (35)^ab^	34 (31)
Responders; ≥30 %	-	-	16^c^	11	12^c^	6	7^c^	2
Responders; ≥50 %	-	-	8	8	7^d^	4	5^d^	1
Pain area	100	100	62.4 (95.9)^e^	76.5 (99.9)	62.9 (105.6)^e^	74.8 (149.1)	44.5 (141.3)^e^	81.2 (189.3)
Physical functioning	*n* = 19	*n* = 19	*n* = 20	*n* = 17	*n* = 16	*n* = 8	*n* = 10	*n* = 5
GCPS								
Grade 0	5	12	8	11	7	6	6	2
Grade I	4	1	6	1	1	1	1	2
Grade II	9	4	4	2	3	1	1	1
Grade III	0	1	1	3	3	0	2	0
Grade IV	1	1	1	0	1	0	0	0
JDC								
Score (0–12)	3 (5)	2 (4)	3 (5)	2 (4.5)	3 (4)	2 (5.5)	3 (5)	1 (3.5)
MVO								
Without pain (mm)	41.1 (9.3)	44.0 (10.9)	42.9 (9.3)	47.6 (9.4)	43.3 (9.4)*	46.9 (9.4)	47.2 (10.8)*	46.1 (6.2)
With pain (mm)	49.7 (7.5)	52.8 (10.0)	47.9 (8.2)	52.8 (9.5)	49.2 (7.5)	51.3 (9.8)	49.3 (10.5)	50.4 (7.5)

The table presents median (IQR) weekly pain intensity (VAS; 0–100 mm), number of responders to treatment (≥30 % and ≥50 % decrease in pain intensity), median (IQR) pain area (%) normalized to baseline, distribution of physical functioning assessed with the Graded Chronic Pain Scale (GCPS) and median (IQR) limitation in jaw function assessed with the Jaw Disability Checklist (JDC) as well as the mean (SD) maximum voluntary mouth opening capacity (MVO) with and without pain

*GRA* Granisetron (Kytril®; 1 mg/mL, Roche, Stockholm, Sweden), *CTR* control substance (isotonic saline (NaCl); 0.9 mg/mL, Fresenius Kabi, Uppsala, Sweden), *IQR* Interquartile range (the 75^th^ percentile minus the 25^th^ percentile), *SD* Standard deviation, *VAS* Visual Analogue Scale, *AU* Arbitrary units

Physical functioning: GCPS: Grade 0 = no disability; Grade I = low disability and low intensity pain; Grade II = low disability and high intensity pain; Grade III = high disability and moderately limiting; Grade IV = high disability and severely limiting

^a^Significant decrease compared to baseline (Dunn’s test: 1 month: *P* = 0.031; 2 months: *P* = 0.008; 6 months: *P* = 0.007. ^b^Significant difference compared to the CTR-group (Mann–Whitney *U*-test; 2-months: *P* = 0.009; 6-months: *P* = 0.031). ^c^Significant difference compared to the CTR-group (*χ* ^2^-test; *P* < 0.001)

^d^Significant difference compared to the CTR-group (*χ* ^2^-test; 2 months: *P* = 0.027; 6 months: *P* < 0.001). ^e^Significant difference (Holm Sidak’s test; 1 month: *P* = 0.039; 2 months: *P* = 0.039; 6 months: *P* = 0.042). *Significant difference compared to baseline (Holm Sidak’s test; *P* < 0.001), but not in the CTR-group

At the 1-month follow-up 80 % of the patients in GRA-group and 55 % in CTR-group reported a reduction of 30 % in weekly pain intensity (ITT-analysis). At the 2-month follow-up these frequencies were 60 % in the GRA-group and 30 % in the CTR-group, and at the 6-month follow-up 35 % and 10 %, respectively. All these frequencies differed significantly between the groups (Table 3).

The frequencies for a reported reduction of 50 % in weekly pain intensity were 40 % in both groups at the 1-month follow-up, 35 % in the GRA-group and 20 % in the CTR-group at the 2-month follow-up, and at the 6-month follow-up 25 % in the GRA-group and 5 % in the CTR-group. These frequencies differed significantly between the groups at the 2- and 6-month follow-ups (Table 3).

The numbers needed to treat (NNT) for a weekly pain reduction of 30 % were 4 at the 1-month follow-up, 3.3 at the 2-month follow-up and 4 at the 6-month follow-up in favor of granisetron (ITT-analysis). The NNT, for a weekly pain reduction of 50 % were 6.7 at the 2-month follow-up and 5 at the 6-month follow-up. The 1-month NNT could not be calculated because of an equal numbers of responders in both groups.

**Fig. 2:**
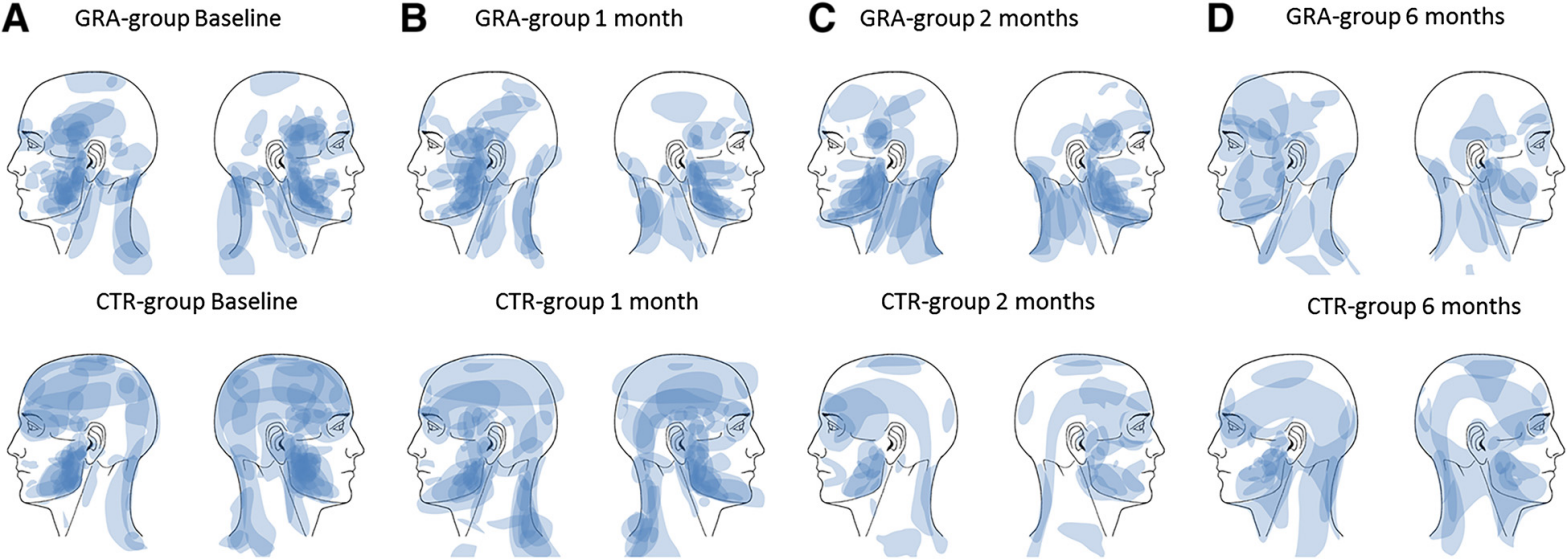
Pain drawings at baseline and follow-ups after treatment with either granisetron or control substance. The pain area at baseline (**a**) and follow-ups (1-month (**b**); 2-months, (**c**); 6-months (**d**)) after treatment with either granisetron (GRA-group) or control substance (isotonic saline; CTR-group) in 40 patients with myofascial temporomandibular disorders. The darker the area is the larger is the overlap from the different participants. The pain area decreased significantly in the GRA-group (Holm-Sidak’s test; *P* < 0.042) compared to baseline but not in the CTR-group (Holm-Sidak’s test; *P* > 0.065)

The pain area had decreased significantly with approximately 40 % at the 1- and 2-months follow-ups, and with 55 % at the 6-month follow-up in the GRA-group. There were no significant changes in the CTR-group (Table 3 and Fig. 2).

### Secondary treatment outcomes

#### Physical functioning

There were no significant differences between the groups in physical functioning according to the GCPS and the JDC, neither at baseline, nor at any of the follow-ups (Table 3).

The MVO increased significantly after treatment in the GRA-group (RM ANOVA; *P* < 0.001, but not in the CTR-group (RM ANOVA; *P* = 0.051). There were no significant differences between the groups at any time point (Table 3). There were no significant changes in MVO with pain within or between groups.

#### Emotional functioning

**Fig. 3:**
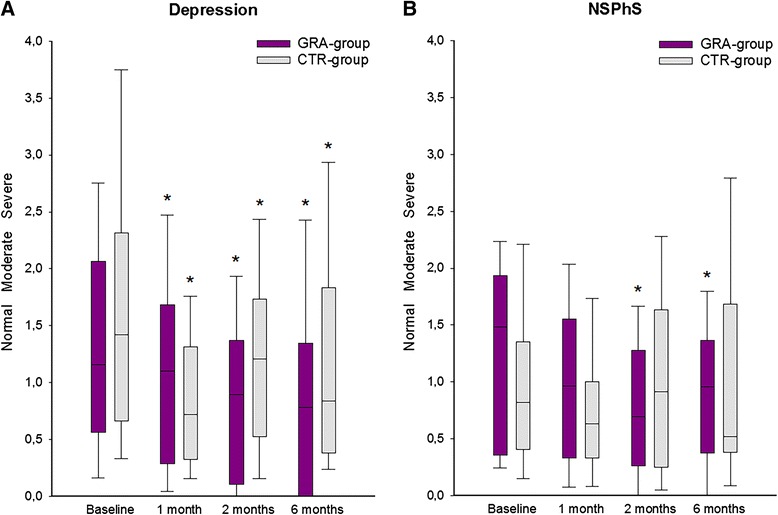
Scores for depression and nonspecific physical symptoms at baseline and follow-ups after treatment. Median (IQR) differences in scores for depression (**a**) and nonspecific physical symptoms (NSPhS) (**b**) in 40 patients with myofascial temporomandibular disorders before (baseline) and after repeated intramuscular tender-point injections with granisetron (GRA-group) or control substance (isotonic saline; CTR-group). Depression-scores are classified as normal (<0.535), moderate (0.535-1.105) and severe (>1.105), and the NSPhS-scores are classified as normal (<0.5), moderate (0.5-1) and severe (>1). There were no significant differences between groups in depression- or in NSPhS-scores at baseline. *Significant difference compared to baseline (Friedman-test with Dunn’s post-hoc test; *P* < 0.05)

At baseline, most patients had moderate to severe scores for depression and NSPhS without group differences. None of the scores differed significantly between responders (30 %, 50 % reduction in pain intensity) and non-responders to any treatment at baseline. The depression-scores decreased significantly over time, in both groups. The NSPhS-scores decreased significantly at the 2-, and 6-month follow-ups in the GRA-group only (Fig. 3).

#### Overall improvement

At the 1-month follow-up the majority of the patients in both groups (GRA-group: 80 % and CTR-group: 78 %) reported an overall improvement of “better”, “much better” or “symptom-free” (ITT-analysis). At the 2-month follow the corresponding frequencies were 81 % and 62 % respectively, and at the 6-month follow-up 90 % in the GRA-group and 60 % in the CTR-group. The differences between groups were significant at the 2- and 6-month follow-ups (Table 4).

**Table 4:** Overall improvement at 1 month, 2 as well as 6 months after repeated tender-point injections with active substance (GRA) or control substance (CTR) in 40 patients with myofascial temporomandibular disorders

	1 month		2 months		6 months	
	GRA (*n* = 20)	CTR (*n* = 18)	GRA (*n* = 16)	CTR (*n* = 8)	GRA (*n* = 10)	CTR (*n* = 5)
Overall improvement						
“No change” to “Worse”	4	4	3	3	1	2
“Better” to “Symptom-free”	16	14	13*	5	9*	3
* “Better”*	*9*	*8*	*6*	*2*	*3*	*1*
* “Much better” or “Symptom-free”*	*7*	*9*	*7*	*3*	*6*	*2*

*GRA* Granisetron (Kytril®; 1 mg/mL, Roche, Stockholm, Sweden), *CTR* control substance (isotonic saline (NaCl); 0.9 mg/mL, Fresenius Kabi, Uppsala, Sweden); *Significant difference compared to the CTR-group (*χ* ^2^-test; 2 months: *P* = 0.005, 6-months: *P* < 0.001)

#### Adverse events

Four patients in both groups reported mild, short lasting adverse events, such as nausea, constipation, dizziness, hematoma and itching after the first injection of substance. These adverse events did not occur after the second and third injections. In the CTR-group one patient reported hives to the first injection and ended the participation, due to assumed allergic reaction.

### Additional treatment outcomes

#### Changes in pressure pain threshold

**Fig. 4:**
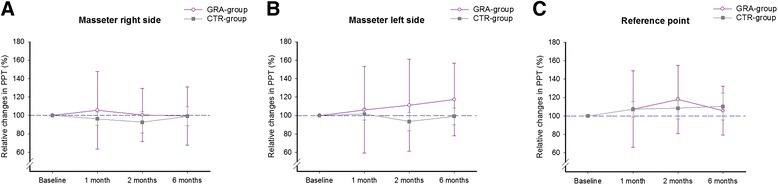
Pressure pain thresholds at baseline and follow-ups after treatment with either granisetron or control substance. The mean (SEM) relative changes (%) of pressure pain threshold (PPT) in 40 patients with myofascial temporomandibular disorders before (baseline) and after repeated intramuscular tender-point injections with granisetron (GRA-group) or control substance (isotonic saline; CTR-group) over the **a** the right masseter muscle **b** the left masseter muscle and **c** over a reference point on the tip of the right index finger. There was no change in PPT after treatment with any of the substances

The PPT over the masseter muscles and the reference point did not differ between groups at baseline and there were no significant differences between the right and left masseter muscles at baseline. Further, PPT did not change over time and there were no differences between substances at any time point (Fig. 4).

#### Correlations between outcome measures and P-5-HT

There were no significant correlations between P-5-HT values and pain or emotional function at baseline. Neither were there any significant correlations between treatment effects regarding pain characteristics or depression- and NSPhS-scores and P-5-HT values at baseline.

## Discussion

To our knowledge this is the first RCT to show that local treatment with a 5-HT_3_ blocker is effective for chronic muscle pain. It was shown that, compared to control, repeated intramuscular tender-point injections with granisetron into jaw muscles of patients with M-TMD had a clinically significant effect on pain intensity and pain area. The effect was still evident 6 months after treatment, when 35 % of the patients in the GRA-group reported a 30 % improvement of pain intensity.

The pain relieving results from this study support previous results that intramuscular injections of 5-HT_3_-receptor antagonists are effective for a number of muscular pain conditions [23, 34]. Human experimental studies have shown that granisetron reduces acute experimental muscle pain intensity, pain area and allodynia induced by 5-HT, hypertonic saline and acidic saline [14, 18, 39]. In patients with fibromyalgia systemic administration of tropisetron (5 mg), reduced pain intensity, tender-point count and improved sleep [19, 20], indicating that 5-HT_3_-receptor antagonists might even be efficacious in more generalized pain conditions, such as fibromyalgia. Although recent studies suggest that peripheral nociceptive inputs, acting as “pain generators”, play an important in maintaining the muscle sensitization in patients with fibromyalgia, indicating that local treatment of these “pain generators” to some extent could decrease pain in patients with fibromyalgia [40], a previous study with a local injection of granisetron into the masseter muscle of patients with fibromyalgia did not show any significant effect on pain or hyperalgesia [41]. However, this study only used a single injection and not repeated tender-point injections as the present and the pain reducing effect was only assessed during 30 min. Thus, there is a need to further investigate if repeated tender-point injections of 5-HT_3_-receptor antagonists might have a pain reducing effect in generalized pain conditions such as fibromyalgia.

5-HT does not only evoke pain directly by 5-HT_3_-receptors, but also interacts with other substances in the chemical milieu around nociceptors. Thus, blocking of 5-HT_3_-receptors may inhibit pain by different mechanisms, which may explain the good analgesic effects. For example, tropisetron is reported to inhibit 5-HT-induced PGE_2_ release from macrophage-like synovial cells in vitro [42] and to have an immunomodulatory function on cytokines [43, 44]. Another reason for its good long-term efficacy might be its route of metabolism by the liver, and that it unlike the other 5-HT_3_-receptor antagonists has a long half-life, and is not dependent on the isoenzyme cytochrome P450 2D6 (CYP2D6), possibly leading to a good efficacy also in ultrarapid metabolizers [31–33].

As many as 81 % (2-month follow-up) and 90 % (6-month follow-up) of the patients in the GRA-group reported an overall improvement of “better”, “much better” or “symptom-free”, which was significantly higher than the CTR-group with 62 % at the 2-month follow-up and 60 % at the 6-month follow-up, which is in line with previous intervention studies with other treatment modalities in chronic M-TMD [45–49].

The NNT in the GRA-group was 3.3 at the 2-month follow-up which is comparable to commonly used analgesics for acute orofacial pain ranging from NNT 1.8 (naproxen, ibuprofen) to NNT 4.5 (aspirin) [50], or topical NSAIDs for acute musculoskeletal conditions ranging from NNT 1.8 (diclofenac) to far over NNT 4 (the majority of topical NSAIDs) [51]. The NNT in the GRA-group was 4.0 at the long-term follow-up, which is better than salicylate-containing topical analgesics for chronic musculoskeletal pain (NNT 6.2) [52] It is also better than botulinum toxin type A for treatment of M-TMD pain that showed NNT values of 11 and 7, after 3 months and 6 months, respectively [53]. Hence, taken together one can consider intramuscularly administrated granisetron as an effective treatment modality for M-TMD.

Previous studies in M-TMD patients have reported improved physical functioning after treatment, shown as lower GCPS severity and JDC-scores [45, 46]. This was not found in this study, although seemingly there was a change towards a lower severity grade in GCPS in the GRA-group. The lack of statistical significance might be due to low statistical power for the secondary outcomes as power calculation was based on pain intensity. However, there was a significant and clinically relevant increase in mouth opening capacity at the 6-months follow-up in the GRA-group indicating an improvement in jaw functioning.

TMD-pain is associated with high levels of depression and NSPhS, even higher than the other TMD diagnoses [54], as was also found in this study. In both groups depression- and NSPhS-scores decreased after treatment and the scores remained decreased over time. This is similar to results from cognitive-behavioral interventions [55] and occlusal appliance therapy [46]. The finding that also the CTR-group showed lower depression-scores is probably due to the decreased pain-intensity.

In both groups serious adverse events were absent, only a few patients reported mild and short-lasting adverse events after the first injections of test substances. Granisetron is considered efficacious and safe. However, systemic administration of 5-HT_3_-blockers is commonly associated with bothersome side effects such as constipation, headache, and dizziness [9, 56]. Intramuscular tender-point injections therefore offers a tempting alternative since no side effects have been reported after local administration [14, 18, 26, 39, 41, 57, 58].

In contrast to several previous experimental studies [17, 18, 41, 57] there was no effect on PPT in this study. One explanation could be that the PPT was assessed the first time one week after each treatment session, whereas the previous studies assessed PPT during 30 min after injection, indicating that any possible effect on PPT is short-lasting. Another reason might be related to sex. In the previous studies increased PPT was mainly found in men [10, 13, 17, 18, 59]. In this study the vast majority of the participants were women, indicating that there might be sex dependent differences.

Although there was a large difference between the GRA-group and CTR-group in mean P-5-HT levels this difference was not significant. Further, there were no correlations between P-5-HT levels and any of the outcome measures or differences between responders and non-responders to treatment. This contrasts a previous study using systemic ondansetron in 21 patients with fibromyalgia, in which the serum 5-HT levels seem to be lower at baseline in the responders [60].

The strengths with this study are the study design, i.e. a prospective, controlled and double-blind RCT performed in a well-characterized patient sample, that the outcome measures recommended by IMMPACT [24, 25] to be used in clinical pain trials were used, and that descriptive data is reported. Hence, data may also be included in future meta-analysis studies. However, even if the results are robust a limited number of patients participated, why the study should be replicated with a larger patient sample. It is also an efficacy study, i.e. conducted in tertiary care. Therefore future studies designed as effectiveness studies in general practice are warranted. The generalizability of the findings should also be investigated by studies in other chronic pain disorders both local and generalized, such as work-related trapezius myalgia or fibromyalgia. Further, there were technical problems with the blood samples. The first 14 (seven from each treatment group) were missing and therefore the results regarding P-5-HT levels must be considered cautiously. Also, one could argue that a limitation of this study is that no consideration was taken regarding the phase of menstrual cycle or the use of contraceptives during the treatment and follow-ups. However, the patients had four consecutive visits one week apart, i.e. during all phases of the menstrual cycle. Further, neither this nor our previous studies have taken the menstrual cycle into consideration since it has been shown that the intra-individual variability in the pain response is greater than the influence of estrogen [61]. Finally, one can discuss the inclusion criteria where chronic pain was defined as pain lasting for more than 3 months and not 6 months which some consider as normal healing phase. However, both 3 and 6 months pain duration is used as time frame in the definition of chronic pain [62, 63], and since no included patient had a duration for less than 12 months this did not affect the outcome of this study.

## Conclusion

In conclusion, the results from the present study show that granisetron has a clinically relevant pain reducing effect, both in a short- and long-term aspect, in patients with chronic M-TMD. Thus, repeated intramuscular injections with granisetron may offer a novel and additional treatment approach for patients with chronic myalgia.

## Abbreviations

TMD: Temporomandibular disorders
M-TMD: Myofascial temporomandibular disorders
TMJ: Temporomandibular joint
5-HT: Serotonin (5-hydroxytryptamine)
PPT: Pressure pain threshold
IMMPACT: the Initiative on Methods, Measurement, and Pain Assessment in Clinical Trials
RDC/TMD: Research Diagnostic Criteria for Temporomandibular disorders
VAS: Visual analogue scale
RCT: Randomized, controlled trial
GRA: Granisetron
CTR: Control
ITT: Intention-to-treat
NNT: Numbers-Needed-to-treat
P-5-HT: Serotonin platelet poor plasma
GCPS: Graded Chronic Pain Scale
JDC: Jaw Disability Checklist
DP: Disability score
MVO: Maximum voluntary mouth opening
NSPhS: Nonspecific physical symptoms
ANOVA: Analysis of variance
